# The role of active brown adipose tissue (aBAT) in lipid metabolism in healthy Chinese adults

**DOI:** 10.1186/s12944-016-0310-8

**Published:** 2016-08-26

**Authors:** Xiaoliang Shao, Wei Yang, Xiaonan Shao, Chun Qiu, Xiaosong Wang, Yuetao Wang

**Affiliations:** Department of Nuclear Medicine, the Third Affiliated Hospital of Soochow University, Changzhou, 213003 China

**Keywords:** Dyslipidemia, aBAT, Chinese, Positron emission tomography, ^18^F-Fluorodeoxyglucose

## Abstract

**Background:**

The prevalence of dyslipidemia in China was increased over the last several years. Studies have shown that the activity of aBAT is related to the lipid metabolism. In this study, we analyzed blood lipid level in tumor-free healthy Chinese adults in order to determine the role of aBAT in lipid metabolism.

**Methods:**

We retrospectively analyzed the factors that affect the blood lipid level in 717 tumor-free healthy adults who received blood lipid measurement and PET/CT scan by multivariate regression analysis. We also determined the role of aBAT on lipid profile by case–control study.

**Results:**

(1) Our results showed that 411 (57.3 %) subjects had dyslipidemia. The prevalence of the subjects with hypercholesteremia, hypertriglyceridemia, low high-density lipoprotein cholesterol and high low-density lipoprotein cholesterol was 9.5 %, 44.4 %, 30.8 % and 1.4 %, respectively. Multivariate logistic regression analysis with dyslipidemia as the dependent variable showed that body mass index (BMI) and smoking are independent risk factors for dyslipidemia (*OR* > 1, *P* < 0.05), while the presence of aBAT is the independent protective factor for dyslipidemia (*OR* < 1, *P* < 0.05). (2) The incidence of aBAT was 1.81 %. Subjects with aBAT had significantly lower serum triglyceride and higher serum high-density lipoprotein cholesterol than the subjects without aBAT. The serum total cholesterol and low-density lipoprotein cholesterol were not significantly different between the subjects with aBAT and those without aBAT.

**Conclusions:**

Dyslipidemia is caused by multiple factors and the presence of aBAT is a protective factor for dyslipidemia in healthy Chinese adults.

## Background

Cardiovascular disease is the primary cause of death in most countries worldwide [1, 2], including China. Dyslipidemia is a major risk factor for atherosclerosis. Dyslipidemia is also the independent risk factor for myocardial infarction [3] and ischemic stroke [4], and therefore represents a serious threat to human health. Due to the low dietary intake of fat and cholesterol, the type of dyslipidemia (mainly hypertriglyceridemia and low high-density lipoprotein cholesterol) in Chinese adult is different from those in Western countries. More importantly, evidence suggests that the prevalence of dyslipidemia in China is significantly increased over the past several years [2, 5].

Brown adipose tissue (BAT) is the main source of non-trembling heat, and it plays an important role in maintaining the body temperature and energy metabolism balance. The mitochondrial inner membrane of brown fat cells is rich in uncoupling protein 1 (UCP l), which converts chemical energy into thermal energy through the uncoupling of oxidative phosphorylation [6]. It was previously believed that BAT exists only in the fetal and infancy period of rodents and humans. However, recent PET/CT studies [7, 8] showed that BAT with high capacity of ^18^F-fluorodeoxyglucose (^18^F-FDG, an analogue of glucose which is used for PET/CT scan) uptake also exists in adult humans, which is recognized as active brown adipose tissue (aBAT). Studies have also shown that the occurrence of aBAT is correlated with age, gender, degree of obesity and outdoor temperature when PET/CT scan was performed.

Most of the previous studies used experimental animals or patients with tumors to investigate the effect of aBAT on lipid metabolism [9–11]. These studies showed that aBAT plays an important role in clearing the triglyceride in the blood. However, cancer patients may have lipid abnormalities when treated with anticancer drugs [12], such as Everolimus, Tamoxifen, Goserelin, which increases the complexity to study the effect of aBAT on lipid metabolism. In this study, we performed multivariate regression analysis to identify the factors that contribute to dyslipidemia in the tumor-free Chinese adults to explore the role of aBAT in dyslipidemia.

## Methods

### Subjects

The subjects receiving ^18^F-FDG PET/CT scan due to elevated tumor markers of unknown reasons, family history of tumors, and suspected contact with carcinogen from October 2010 to December 2015 were obtained from our Medical Center. For the present analysis, the inclusion criteria were as follows: 1) adult (older than 18 years), 2) no history of cancer, 3) receiving blood lipid measurement at the same day when ^18^F-FDG PET/CT scans were performed. The exclusion criteria were: 1) subjects who had been diagnosed as dyslipidemia and took oral lipid-lowering drugs, 2) subjects who took oral β-receptor blockers, 3) subjects who took hormone drugs (such as thyroid hormone, estrogen, steroids), 4) subjects who had acute and chronic liver and kidney dysfunction, 5) subjects who had disorders that may lead to malabsorption (such as chronic atrophic gastritis, anorexia, etc.).

A total of 717 subjects met the inclusion criteria and we recorded the gender, age, height, weight, body mass index (BMI), smoking status (not including passive smoking), alcohol (drinking at least once a week), fasting plasma glucose concentrations, blood lipid levels (including total cholesterol (TC), triglycerides (TG), high-density lipoprotein cholesterol (HDL-C), low-density lipoprotein cholesterol (LDL-C)), and the outdoor temperature of the day when PET/CT scans were performed. According to Chinese control of overweight and obesity guidelines [13], subjects with BMI between 24 and 27.9 kg/m^2^ are considered overweight, and subjects with BMI ≥ 28 kg/m^2^ are considered obese. According to the American Diabetes Association guidelines [14], subjects with the fasting plasma glucose concentration ≥ 7.0 mmol/L or subjects who were previously diagnosed with diabetes and are being treated are considered as diabetic.

### Blood lipid analysis and criteria for dyslipidemia

Blood samples (5 ml, obtained after minimal 8 h of fasting) were collected before ^18^F-FDG PET/CT scan. The concentrations of serum TC, TG, HDL-C and LDL-C were measured by enzymatic methods using Hitachi 7600–120 automatic biochemical analyzer. Internal quality control was performed at the same time and all operations were in strict accordance with the instructions. Subjects with at least one of the following criteria: serum TC ≥ 6.22 mmol/L (240 mg/dl), serum TG ≥ 2.26 mmol/L (200 mg/dl), serum HDL-C < 1.04 mmol/L (40 mg/dl), and serum LDL-C ≥ 4.14 mmol/L (160 mg/dl) can be considered as dyslipidemia. Factors, such as age, sex, BMI, smoking, drinking, diabetes, and the presence of aBAT that may have an impact on blood lipids were analyzed.

### PET/CT scan and definition of aBAT

PET/CT scan was performed using ^18^F-FDG (radiochemical purity > 95 %) as the imaging agent (Siemens Biograph mCT (64) type PET/CT instrument). Before PET/CT scan, the subjects underwent minimal 8 h of fasting. Subjects with the fasting plasma glucose below 11.1 mmol/L were intravenously injected with ^18^F-FDG at the average dose of 4.74 ± 0.93 MBq/kg. Following 45–60 min of rest in a quiet, warm and dark environment and urination, PET/CT scan was performed. Syngo Ture D system was used for image reconstruction, which forms cross-sectional, coronal, sagittal tomographic image and three-dimensional projection image.

**Fig. 1:**
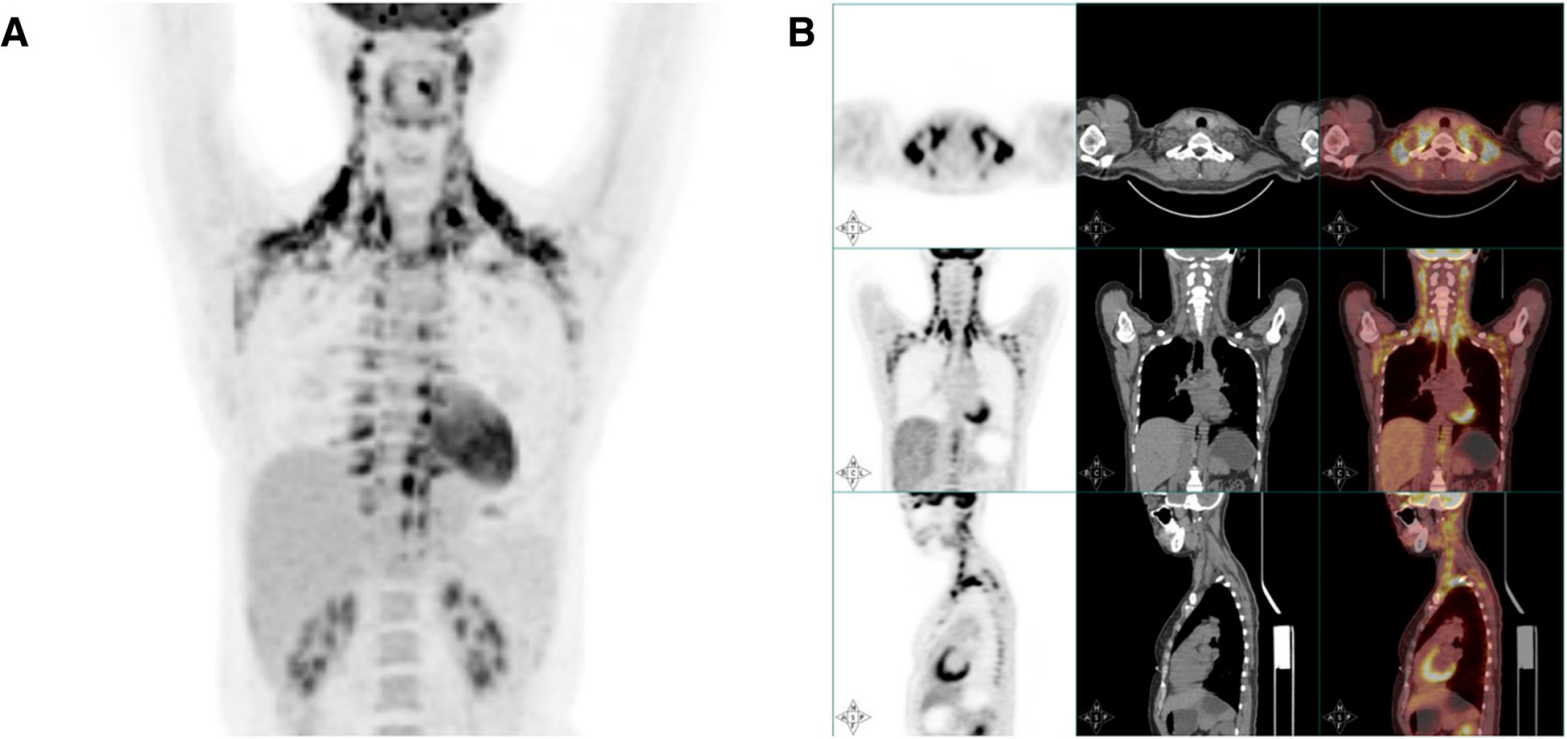
(**a** and **b**) Distribution of aBAT in one subject included in this study

According to the literature [7], tissues that are located at the neck and supraclavicular region, mediastinum and both armpits, on both sides of the spine, kidney and other peripheral regions are considered as aBAT if CT value is at the adipose tissue density (CT value of −250 ~ −50HU), the diameter is larger than 4 mm, and maximum standardized uptake value of ^18^F-FDG in PET imagings is at least 2 (SUVmax ≥ 2) (Fig. 1). Active BAT was detected in 13 subjects with an occurrence of 1.8 % (13/717). To reduce the impact of sampling error and to observe the effect of aBAT on blood lipids more intuitively, 1:3 ratio between the number of the subjects in aBAT group and that in control group was used because 1:3 or 1:4 ratio can obtain higher statistics power [15]. The gender, age, BMI, fasting blood glucose and the outdoor temperature were not significantly different between the two groups. In order to reduce subjective bias, lipid profiles were not considered when the control group was selected.

One subject (34 years old) was a female with a BMI of 20.2 kg/m^2^ and fasting blood glucose of 5.0 mmol/L. Due to a family history of tumor, the subject requested ^18^F-FDG PET/CT whole body scan. The outdoor temperature at the day when the scan was performed was 8 °C. Active BAT was distributed on both sides of neck, armpits, supraclavicular region, mediastinum, and the spine.

### Statistical analysis

Statistical analyses were performed using SPSS 23.0 software. Measurement data were expressed as mean ± standard deviation (± s). Kolmogorov-Smirnov test was first performed to determine whether the measurement data follow a normal distribution. The normally distributed data were compared using independent *t*-test. The non-normally distributed data were compared using Mann–Whitney u rank sum test. The percentage data were compared using *χ*^2^ test. When the theoretical frequency in a linked list was less than 1, Fisher exact test was used. Logistic regression analysis with two categorical variables was performed to determine the factors that can affect dyslipidemia. The regression coefficient, odds ratio (OR) and 95 % confidence intervals were calculated. *P* <0.05 (two-sided) was considered statistically significant.

## Results

### Characteristics of the subjects

The general characteristics of studied subjects are listed in Table 1.

**Table 1:** Baseline characteristics of studied subjects

Parameters	Subjects (*n* = 717)
Age (years)	49.8 ± 9.7
Male (%)	66.4
BMI (kg/m^2^)	24.8 ± 3.2
Normal weight (%)	39.8
Overweight (%)	45.6
Obesity (%)	14.6
Current Smoker (%)	38.4
Current Drinker (%)	34.3
Diabetes (%)	12.7
Outdoor temperature (°C)	21.6 ± 9.1 °C
aBAT (%)	1.81

*BMI* body mass index, *aBAT* active brown adipose tissue

Normal weight: BMI < 24 kg/m^2^, Overweight: BMI between 24 and 27.9 kg/m^2^, Obesity: BMI ≥ 28 kg/m^2^

Outdoor temperature was the outside temperature of the day when PET/CT scan was performed

### Univariate and multivariate logistic regression analysis of dyslipidemia

Among 717 subjects included in this study, 411 subjects (57.3 %) had dyslipidemia. The average serum TC, TG, HDL-C, and LDL-C levels were 5.20 ± 1.08 mmol/L, 3.57 ± 2.55 mmol/L, 1.08 ± 0.25 mmol/L, and 2.52 ± 0.69 mmol/L, respectively. The percentages of the subjects with hypercholesteremia, hypertriglyceridemia, low HDL-C and high LDL-C were 19.5 %, 44.4 %, 30.8 % and 1.4 %, respectively. Lipid level was designated as variable (abnormal y = 1, normal y = 0), and univariate analysis was performed to identify factors (age, sex, BMI, smoking, drinking, diabetes, aBAT) that may affect dyslipidemia (Table 2). The statistically significant factors in univariate analysis were further analyzed by multivariate logistic regression analysis. These results showed that high BMI and smoking were independent risk factors for dyslipidemia (*OR* > 1, *P* < 0.05), and the presence of aBAT was an independent protective factor for dyslipidemia (*OR* < 1, *P* < 0.05) (Table 3).

**Table 2:** Univariate analysis of factors contributing to dyslipidemia

	Dyslipidemia (*n* = 411)	Normal (*n* = 306)	*P* value
Age (years)	49.8 ± 9.4	49.9 ± 10.1	0.942
Male (%)	74.2	55.9	<0.001^*^
BMI (kg/m^2^)	25.5 ± 2.9	23.8 ± 3.2	<0.001^*^
Normal weight (%)	30.7	52.0	<0.001^*^
Overweight (%)	49.9	39.9	
Obesity (%)	19.4	8.1	
Current Smoker (%)	45.7	28.4	<0.001^*^
Current Drinker (%)	38.2	29.1	0.011^*^
Diabetes (%)	15.3	9.2	0.014^*^
aBAT (%)	0.2	3.9	<0.001^*^

*BMI* body mass index, *aBAT* active brown adipose tissue

Normal weight: BMI < 24 kg/m^2^, Overweight: BMI between 24 and 27.9 kg/m^2^, Obesity: BMI ≥ 28 kg/m^2^

^*^Significant difference

**Table 3:** Multivariate and Logistic regression analysis of factors contributing to dyslipidemia

Variables	Regression coefficient	*OR*	95 % *CI*	*P value*
Gender	−0.271	0.762	0.497 ~ 1.170	0.215
BMI	0.162	1.175	1.112 ~ 1.234	<0.001^*^
Smoke	0.521	1.684	1.134 ~ 2.501	0.010^*^
Drink	−0.158	0.854	0.577 ~ 1.265	0.432
Diabetes	0.236	1.266	0.769 ~ 2.085	0.353
aBAT	−2.575	0.076	0.010 ~ 0.610	0.015^*^

*OR* odds ratio, *CI* confidence index, *aBAT* active brown adipose tissue

*Significant difference

### Comparison of the blood lipids between the aBAT group and the control group

**Fig. 2:**
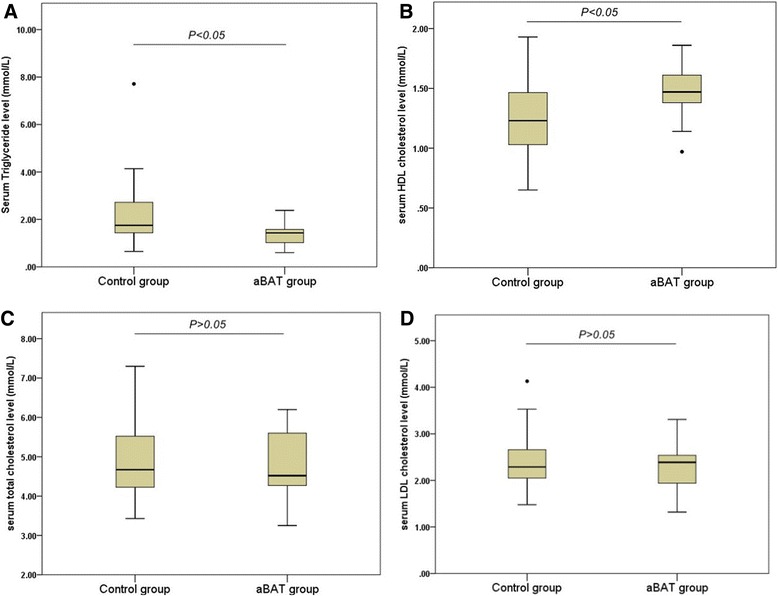
Comparison of the blood lipid level between aBAT and control groups. Comparison of blood TG between aBAT and control groups (**a**); Comparison of blood HDL-C between aBAT and control groups (**b**); Comparison of blood TC between aBAT and control groups (**c**); Comparison of blood LDL-C between aBAT and control groups (**d**)

The 13 subjects with the presence of aBAT were defined as aBAT group. The aBAT-negative subjects with similar age, gender ratio, BMI, fasting glucose level and outdoor temperature were selected as control group (Table 4). The levels of TC, TG, HDL-C and LDL-C were compared between the two groups. The results showed that serum TG level in aBAT group was significantly lower than that in the control group (1.41 ± 0.54 vs. 2.70 ± 2.88 mmol/L, *P* = 0.024). The level of serum HDL-C in the aBAT group was significantly higher than that in the control group (1.46 ± 0.26 vs. 1.26 ± 0.30 mmol/L, *P* = 0.032). The levels of serum TC and LDL-C in aBAT group were lower (but not significantly) than those in the control group (TC: 4.79 ± 0.93 vs. 4.96 ± 1.0 mmol/L and LDL-C: 2.28 ± 0.61 vs. 2.36 ± 0.52 mmol/L) (*P* = 0.587 and *P* = 0.620, respectively) (Fig. 2A-2D). The ratio TC/HDL-C in the aBAT group was significantly lower than that in the control group (3.36 ± 0.78 vs. 4.19 ± 1.40 mmol/L, *P* = 0.047).

**Table 4:** Characteristics of subjects in aBAT and control groups

	aBAT group (*n* = 13)	Control (*n* = 39)	*P* value
Age (years)	41.9 ± 6.5	41.8 ± 6.4	0.950
Male /Female	5/8	15/24	
BMI (kg/m^2^)	22.9 ± 2.6	23.7 ± 2.3	0.286
Fasting plasma glucose (mmol/L)	5.08 ± 0.59	5.45 ± 0.60	0.062
Outdoor temperature (°C)	12.5 ± 7.1	12.7 ± 7.3	0.947

*aBAT* active brown adipose tissue, *BMI* body mass index

Outdoor temperature was the outside temperature of the day when PET/CT scan was performed

## Discussion

Dyslipidemia often includes hypercholesteremia, hypertriglyceridemia, high LDL-C and low HDL-C [16]. The types of dyslipidemia in Asian countries including China are mainly manifested as hypertriglyceridemia and low HDL-C, which is different from those in the United States and Europe [2, 5, 17]. In this study, the occurrence of dyslipidemia was 57.3 %, with the incidence of hypertriglyceridemia and low HDL-C at 44.4 % and 30.8 %, respectively, which is slightly higher than the recent epidemiological findings [5], but basically reflects the abnormal status of blood lipids in this region. PET/CT scan showed that the occurrence of aBAT in 717 subjects was 1.8 %, which was similar to that in other neighboring cities with similar latitude. Zhang et al. [18] showed that aBAT occurrence was 1.58 % in 31,088 subjects receiving PET/CT scan.

Univariate analysis showed that dyslipidemia was correlated with multiple factors including gender, BMI (overweight and obesity), smoking, alcohol consumption, and diabetes, suggesting that dyslipidemia is not caused by a single factor, but rather caused by multiple factors. Furthermore, multivariate regression analysis showed that BMI and smoking are independent risk factors for dyslipidemia, which is consistent with the results of previous studies [19, 20]. More importantly, we found that aBAT is a protective factor for dyslipidemia in the healthy Chinese adults (OR = 0.076, *P* < 0.05).

There were relatively few clinical studies examining the effect of aBAT on lipid metabolism. Zhang et al. [21] showed that aBAT was positive in 62 out of 3329 subjects receiving PET/CT scans. Furthermore, HDL-C is higher, while TC/HDL-C is lower in the subjects with positive aBAT compared to the subjects with negative aBAT. However, in that study, BMI was significantly different between aBAT-positive group and the aBAT-negative group. Studies have demonstrated that BAT level and activity were negatively correlated with BMI [7]. Van Marken et al. [22] showed that subjects with BMI of 38.7 kg/m^2^ did not exhibit BAT imaging even the subjects were exposed to a cold environment. In a retrospective study of 5,907 cases of cancer patients (Caucasian), Ozguven et al. [11] showed that 25 subjects were aBAT-positive with a positive rate of 0.4 %. Based on the 1:3 ratio between the number of subjects in aBAT-positive group and that in the control group, the study showed that blood TC and LDL-C in the aBAT-positive group were significantly lower than that in the control group. However, blood TG and HDL-C were not significant different between the two groups. These findings suggested that different population (e.g., physical exam group, tumor group), or population in different regions had different types of dyslipidemia (Chinese population mainly had high TG and low HDL-C, while Europeans and Americans mainly had high TC and high LDL-C). The effect of aBAT on different lipid components is different. The results of this study were partially consistent with previous studies [21]. However, we also showed that serum TG level in aBAT group was lower than that of the control group, which is different from other studies [11, 21].

Animal studies [10, 23] have indicated that increasing BAT activity can increase TG clearance in plasma. Bartelt et al. [23] found that increasing the BAT activity in vivo in mice by cold stimulation can modulate the clearance of triglyceride-rich lipoproteins (TRLs), resulting in decreased levels of serum TG and increased levels of HDL-C. This process is dependent on the increased vascular endothelial permeability to lipoprotein, local lipoprotein lipase activity and transmembrane receptor CD36. Khedoe et al. [10] found that BAT takes up plasma TG preferentially by means of lipolysis-mediated uptake of fatty acids. These animal studies revealed the mechanisms of low TG and high HDL-C in the aBAT group in this study. These studies also demonstrated the important value of aBAT in lipid metabolism.

Statins are the most widely used drugs to treat hyperlipidemia and can reduce the cholesterol concentration by 30 % [24]. However, statin treatment only prevents 25 % to 45 % of all cardiovascular events [25]. One potential factor limiting further reduction in cardiovascular events is the residual elevation in serum TG levels [26]. aBAT was demonstrated as a key player in triglyceride metabolism in our study. Furthermore, aBAT decreases cholesterol levels as well [9, 11]. Our results provide a new therapeutic target to prevent hyperlipidemia and atherosclerosis.

### Limitations

The study was not a large-scale multicenter epidemiological survey and the occurrence of dyslipidemia only reflects the area that we studied. The occurrence of aBAT is similar to other cities at the same latitude [18]. However, only 717 subjects met the inclusion criteria and there were only 13 aBAT-positive subjects. Although we have strict control by setting the standard and blind selection (concealing the status of subjects in the control group and hyperlipidemia during selection), subjective factors leading to the selection bias cannot be completely avoided.

## Conclusions

Dyslipidemia is not caused by a single factor. In contrast, it is the result of multiple factors. This study demonstrated that obesity and smoking are risk factors for dyslipidemia, and a healthy lifestyle is the basis for the prevention and control of dyslipidemia. More importantly, this study demonstrated that aBAT has a protective effect against dyslipidemia, which is mainly manifested by lower levels of serum TG and TC/HDL-C, and higher HDL-C level in the aBAT group. Clinical studies have verified that aBAT can increase plasma TG clearance, indicating the value of aBAT the prevention of dyslipidemia and cardiovascular and cerebrovascular diseases. At the same time, it provides a new target for the development of lipid-lowering drugs.

## Abbreviations

^18^F-FDG: ^18^F-fluorodeoxyglucose
aBAT: Active brown adipose tissue
BAT: Brown adipose tissue
BMI: Body mass index
HDL-C: High-density lipoprotein cholesterol
LDL-C: Low-density lipoprotein cholesterol
OR: Odds ratio
TC: Total cholesterol
TG: Triglycerides
UCP l: Uncoupling protein 1
